# New Diagnostic and Staging Framework Applied to Established PD in the BioFIND Cohort

**DOI:** 10.21203/rs.3.rs-6003205/v1

**Published:** 2025-04-28

**Authors:** Marco J. Russo, Un Jung Kang

**Affiliations:** Rutgers University, Robert Wood Johnson Medical School; NYU Grossman School of Medicine

## Abstract

The proposed Neuronal α-Synuclein Disease Integrated Staging System (NSD-ISS) was recently applied to early Parkinson’s disease (PD) cohorts. We applied this research framework to the BioFIND study cohort, which includes more moderately advanced PD participants with clinically established PD. Disease durations within each ISS stage were highly variable. Cognitive and non-motor anchors had little weight in determining staging. The analysis highlights strengths and limitations of NSD-ISS to guide further refinement of an integrated staging system.

## Introduction

Novel methods for antemortem detection of α-synuclein pathology are motivating reappraisal of the challenging clinical heterogeneity of Parkinson disease (PD), and may stimulate new directions toward understanding its pathophysiology. New criteria are proposed that aim to consolidate PD diagnosis according to biological anchors, and thereby better characterize disease trajectory.^1,2^ One such framework for definition and staging of PD, the Neuronal α-Synuclein Disease Integrated Staging System (NSD-ISS), was recently applied to existing databases of PD participants from 3 studies of early mild PD — PPMI, PASADENA, and SPARK — to explore and validate this proposed framework.^3^ Here, we complement their analysis by applying the NSD-ISS system to the BioFIND (Fox Investigation for New Discovery of Biomarkers in Parkinson’s Disease) study, a multi-center, cross-sectional biomarker study of established, moderately advanced PD.^4^ BioFIND includes established PD participants who have had disease for more than 5 years and up to 18 years since symptom onset and have demonstrated levodopa responsiveness, and this represents a moderate PD cohort that is relatively more advanced than the early or *de novo* PD cohorts recently assessed.^3^

BioFIND comprises data from 119 PD participants with disease duration of 6.8 ± 3.5 years (mean ± S.D.) from time of diagnosis.^4^ From these 119 participants, 108 have CSF α-synuclein seed amplification assay (αSyn-SAA) results ('Evaluable'). These 108 participants each had CSF αSyn-SAA performed by 3 independent laboratories.^5^ We restricted analysis to those participants who had positive(+) αSyn-SAA in at least 2 of the 3 independent assays, minimizing the likelihood of including participants with false positive αSyn-SAA results. There were 104 (96.3%) αSyn-SAA+ or S+ participants meeting criteria for neuronal α-synuclein biomarker by the NDS-ISS schema and also for the presence of pathological α-synuclein by SynNeurGe. These 104 participants are therefore 'NSD Stageable', and are at least stage 2A by NSD-ISS. Note, dopaminergic imaging is not available for BioFIND — as these participants have unequivocally manifest motor signs (MDS-UPDRS Part 3 average score is 26.1 ± 12.6 when on medication) and good response to levodopa, we might assume that they are at least stage 2B, but to avoid the explicit assumption, we simply refer to them as minimally stage 2.

**Figure 1:**
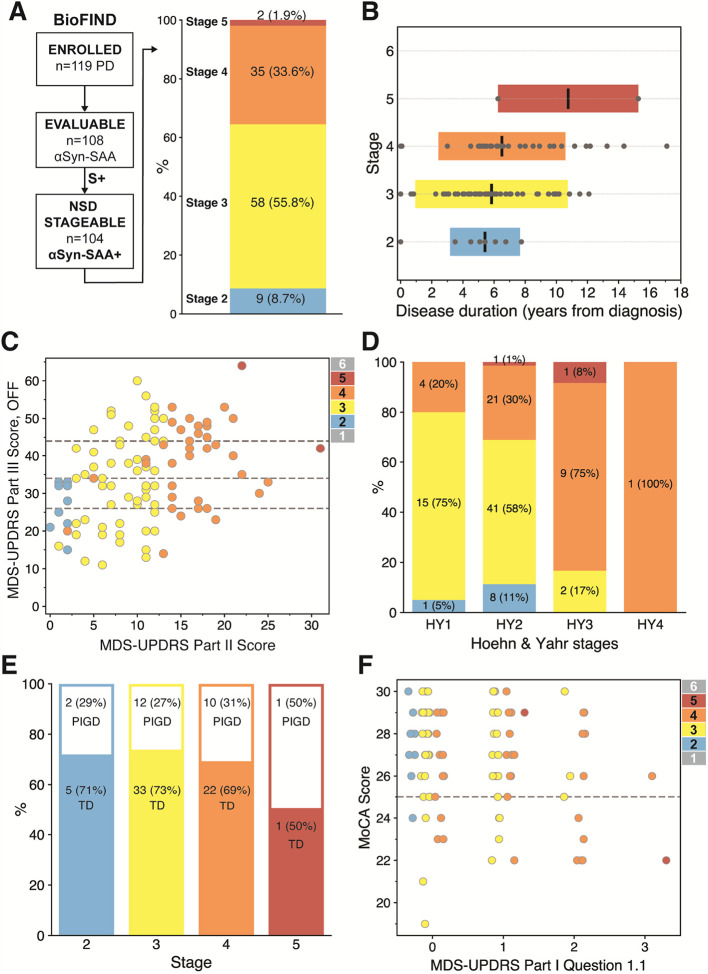
NSD-ISS Applied to BioFIND. (A) Selection of BioFIND participants with evidence of α-synuclein as biological anchor through CSF α-synuclein seed amplification assay (αSyn-SAA). These 104 S+ participants were stageable by NSD-ISS criteria. Single bar plot shows the number and percentage of S+ participants within each clinical stage. Again, no dopamine imaging is available within BioFIND, so these participants are minimally stage 2A and likely stage 2B, but we did not make this distinction. (B) Duration of disease, defined as time since diagnosis, for all participants, within each clinical stage. Each marker is a single participant. Black bars represent mean, and colored blocks indicate standard deviation (stage 2: 5.4 [2.3]; stage 3: 5.8 [4.9]; stage 4: 6.5 [4.1]; stage 5: 10.8 [4.5]; median [IQR], non-parametric Kruskal-Wallis H-statistic 87.5, p=0.44) (C) MDS-UPDRS Part III score when off of motor medications (OFF) is plotted versus MDS-UPDRS Part II scores for each S+ participant – each point represents single participant. NSD-ISS stages are represented by color with key provided in legend. Note the variability of MDS-UPDRS Part III OFF scores within each stage, and overlap across stages. Dotted lines indicate the 25^th^, 50^th^ (median), and 75^th^ percentiles of UPDRS Part III OFF scores. (D) Bar plots indicating distributions of NSD-ISS stages within each Hoehn & Yahr (HY) stage within BioFIND (percentages indicate fraction of each NSD-ISS stage within each HY stage). (E) Fraction of tremor-dominant (TD) and postural instability & gait difficulty (PIGD) motor phenotypes within each NSD-ISS stage within BioFIND. (F) Plot of Montreal Cognitive Assessment (MoCA) score versus MDS-UPDRS Part I Question 1.1 score (Q1.1) for each BioFIND participant, with stages indicated by color. Dotted line indicates MoCA score = 25, which is the typical delineation of mild cognitive impairment and is the demarcation of cognitive progression in the NDS-ISS. Points are 'jittered' from the integral ordinate for visibility.

After applying the integrated staging system to baseline clinical data for 104 S+ participants, the majority of participants were Stage 3 (n=58, 55.8%) similar to those observed for PPMI, PASADENA, and SPARK.^3^ However, more subjects were Stage 4 (n=35, 33.6%), while less were stage 2 (n=9, 8.7%), compared to the early PD cohorts. Interestingly, only 2 (1.9%) were stage 5 and no participants met criteria for stage 6 despite BioFIND participants having considerably longer time since diagnosis than other cohorts (Figure 1A). That ~90% of participants were within stages 3 and 4 implies that participants are approximately normally distributed across stages, rather than uniformly distributed. It is worth considering that a more strategic staging system for some clinical trial designs would have participants uniformly distributed across stages, which would facilitate detection of meaningful changes as participants move from one stage to the next.

Though BioFIND is a cross-sectional study, it includes participants with a wide range of disease durations. We therefore looked at the distribution of disease durations (time from diagnosis) within each NSD-ISS stage (Figure 2B). There were not significant differences in disease duration among the stages (Kruskal-Wallis H-statistic 87.5, p=0.44). Even within later stages, disease duration varies widely, with some designated as stage 4, even at time of diagnosis. This is not explained by variability in the time to diagnosis, as a similar distribution within each stage is seen when time since symptom onset is used (not shown). Many participants with PD for upwards of 10 years did not qualify as stage 6 or even stage 5, which suggests that the timescales of advancement to these stages are unlikely to be relevant on the time frame of practical therapeutic research trials. Participants in later stages may rapidly reach, or have already reached, major disease milestones, as suggested by the Dam *et al.* analysis,^3^ but staging itself does not intrinsically capture the duration of disease. Similarly, if we plot MDS-UPDRS Part III scores (off medication) vs. MDS-UPDRS Part II scores for each participant, we note relatively weak correlation (R^2^=0.14) between these objective (Part III) and subjective (Part II) motor measures (Figure 1C). By highlighting NSD-ISS determined for each participant, we see that NSD-ISS separates stages predominantly according to Part II scores as expected, but with significant variability within stages and overlap across stages for Part III data, particularly for stages 3 and 4. We also looked at distributions of NSD-ISS stages within each Hoehn & Yahr (HY) stage, as HY stages > 2 are associated with greater functional disability and reduced quality of life.^6^ However, the distribution here implies that a subject could move from HY1 to HY4 but remain NSD-ISS stage 4 (Figure 1D). Especially within NSD-ISS stages 3 and 4, these NSD-ISS stages may not provide sufficient resolution by current criteria to capture many clinically meaningful changes that would drive progression across the manifest clinical stages.

Since BioFIND and PPMI data were used to establish the prevalence of distinct motor phenotypes in PD^7^, we examined the relationship of tremor-dominant (TD) and postural instability and gait difficulty (PIGD) phenotypes to NSD-ISS within BioFIND. Within the S+ population, 61 (58.6%) were TD and 25 (24.0%) were PIGD, comparable to the complete BioFIND cohort (Figure 1E).^7^ TD and PIGD forms are present in the same ratio in stages 2-4, suggesting that no stage is preferentially enriched in either phenotype. This is somewhat remarkable when considering that the NSD-ISS largely de-emphasizes objective motor measures in favor of *subjective* motor metrics, and when considering the variability of Part III scores (Figure 1C). We do note that MDS-UPDRS Part III and levodopa equivalent daily dose (LEDD) are generally higher with increasing NSD-ISS stage (Table 1).

The NSD-ISS incorporates existing measures of cognitive impairment, which can potentially determine the stage of patients independently of motor and non-motor symptom severity. By scoring below 25 on the MoCA, for instance, or with cognitive dysfunction that is progressively more limiting to daily activities and social interactions, as indicated by MDS-UPDRS Part I Question 1.1, patients will move further along the integrated stages. We assessed whether the cognitive criteria were meaningful among BioFIND participants. First, it should be noted that only one participant (1 of 104, 0.9%) had his/her stage determined exclusively by the cognitive score (Stage 5 by cognitive criteria alone, but Stage 4 by solely motor or non-motor criteria). The majority of participants' stages were determined by motor criteria, with non-motor or cognitive criteria that were equal or less in severity than motor staging. We examined the distribution of cognitive staging metrics across the stages by plotting MoCA score vs. MDS-UPDRS Part I Question 1.1 (Q1.1) response. Even within the relatively advanced BioFIND cohort, the minimum MoCA score was 19, and the maximum Q1.1 was 3. There is considerable overlap of the various stages across MoCA scores and Q1.1 scores, suggesting that cognitive criteria do not provide informative distinction among these PD participants (Figure 1F). Aside from the rare participant described above, cognitive criteria do not seem to drive NSD-ISS staging of the moderate-to-severe PD phenotypes in BioFIND. Alternative cognitive scales (e.g., PD-CRS, detailed neuropsychologic profiles) could potentially provide additional information to further stratify those participants with intermediate MoCA and Q1.1 scores.

Both the NSD-ISS^1^ and the SynNeurGe^2^ provide a contemporary reassessment of Parkinson disease diagnosis in the era of *in vivo* α-synuclein detection. A biological anchor is established for 96.3% of BioFIND participants, which produces an S+ enriched cohort for further analysis. The NSD-ISS additionally proposes a staging framework to generate distributions of clinical stages of PD that are consistent across multiple study cohorts. However, even within this more advanced PD cohort of BioFIND, the later stages are under-represented, and there is bias towards earlier stages 3 and 4. We regard BioFIND as a clinically more advanced cohort of established PD in comparison with *de novo* cohorts, but there were some selection parameters that could have prevented inclusion of the most advanced or rapidly progressive PD patients. For instance, although BioFIND did not exclude subjects based on MoCA score, it excluded subjects with severe dysautonomia to exclude atypical parkinsonism such as multiple system atrophy. Dysautonomia in PD is linked to both worse motor and cognitive phenotypes.^8^ Also, by aiming to select a relatively homogeneous motor phenotype of PD participants with higher diagnostic specificity to exclude atypical parkinsonism, BioFIND may have excluded more atremulous or akinetic-rigid patients, phenotypes associated with more rapidly progressive forms of the disease.^9^ The BioFIND cohort is slightly less severe compared to subjects tested for advanced symptomatic therapies for motor fluctuations ^10,11^, perhaps reflecting the nature of observational biomarker studies. Of note, most of these advanced therapeutic trials included subjects with minimal cognitive deficit. Nonetheless, the NSD-ISS could be strengthened by modifying criteria to better incorporate non-motor symptoms at earlier stages. The combined effects or weights of cognitive dysfunction, other non-motor symptoms, and motor symptoms that have been shown to have significant impact on overall progression and prognosis – as in the PIGD phenotype, for instance – are under-represented by the proposed staging criteria that emphasizes primarily subjective MDS-UPDRS items. Furthermore, analysis of existing longitudinal data or new longitudinal studies of the same S+ participants over years, and that incorporates clinical milestones, will be essential for better insight into the utility of this staging system to track progression.

## Methods

BioFIND (Fox Investigation for New Discovery of Biomarkers in Parkinson's Disease) is an observational clinical study designed to discover and verify biomarkers of Parkinson's disease (PD). The study was carried out at eight academic sites in the United States. Recruitment began in 2012 and closed in March 2015. BioFIND collected clinical data and biospecimens, including blood and cerebrospinal fluid, in 118 well-defined, moderately advanced people with PD and 88 control volunteers. Enrolled PD subjects met the United Kingdom PD Society Brain Bank (UKPDBB) clinical diagnostic criteria, modified to require all three classic motor signs of parkinsonism (i.e., bradykinesia, rigidity, and resting tremor), by history or examination, instead of just two signs (bradykinesia with either rigidity, resting tremor, or postural instability). Informed consent was obtained from all participants, and all study protocols were approved by the institutional review boards for the University of Rochester Clinical Trials Coordination Center (CTCC) and individual sites. Clinical and biomarker data from the BioFIND study were accessed through the Laboratory of Neuro Imaging (LONI) Image & Data Archive database (ida.loni.usc.edu). All data exploration and analysis were performed with Python-based tools (Python 3.12.2, Pandas 2.2.2), statistical analysis with Scikit-learn 1.5.1 (linear regression), and figures were generated with combination of Matplotlib 3.8.4 and Seaborn 0.13.2. Documentation and code are available at www.github.com/dr-russo/nsd-iss_biofind.

## Figures and Tables

**Table 1:** Properties of BioFIND moderate-to-advanced PD cohort by NSD-ISS stage

		Stage				
		2 n=9	3 n=58	4 n=35	5 n=2	All S+ n=104
Age		66.4 (6.9)	67.9 (6.7)	69.1 (6.3)	68.6 (5.5)	68.2 (6.5)
Gender (M/F)		5/4	35/23	19/16	2/0	61/43
Disease duration (years)		5.1 (2.3)	6.4 (2.9)	7.4 (3.6)	10.8 (6.4)	6.7 (3.3)
MDS-UPDRS	Part I Total	3.4 (3.3)	7.1 (3.0)	12.5 (5.5)	14.5 (3.5)	8.7 (5.0)
	Part II Total	1.4 (0.7)	8.6 (3.2)	16.1 (4.5)	26.5 (6.4)	10.8 (6.1)
	Part III ON Total	19.3 (7.8)	24.5 (12.3)	29.3 (13.1)	44.0 (7.1)	26.1 (12.7)
	Part III OFF Total	26.8 (6.4)	33.8 (12.5)	37.9 (10.3)	53.0 (15.6)	35.0 (11.9)
LEDD		737 (478)	685 (510)	869 (752)	1286 (392)	764 (603)
MoCA Total		27.4 (1.7)	27.2 (2.4)	26.2 (2.4)	25.5 (4.9)	26.8 (2.4)
TD Phenotype, n (%)		5 (56%)	33 (57%)	22 (63%)	1 (50%)	61 (59%)
PIGD Phenotype, n (%)		2 (22%)	12 (21%)	10 (29%)	1 (50%)	25 (24%)

Mean (S.D.), unless indicated

## Data Availability

Data used in the preparation of this article were obtained from the Fox Investigation for New Discovery of Biomarkers (BioFIND) database. For up-to-date information on the study, visit www.michaeljfox.org/biofind.

## Code Availability:

All code used for cleaning and analysis of data in this study is publicly available on Github at www.github.com/dr-russo/nsd-iss_biofind.

## Consortia

The Fox Investigation of New Biomarker Discovery (BioFIND) Study Author Information

Un Jung Kang^2^, Roy N. Alcalay^3^, Amy Amara^4^, Vanessa Arnedo^5^, Samuel Frank^6^, Mark Frasier^5^, Jennifer G. Goldman^7^, Katrina Gwinn^8^, Claire Henchcliffe^9^, Penelope Hogarth^10^, Anna Naito^5^, Alyssa N. Reimer^5^, Marie-Helene Saint-Hilaire^11^, Christine Swanson-Fischer^8^, Margaret Sutherland^8^, Paul Tuite^12^, Tao Xie^13^

^3^ Division of Movement Disorders, Department of Neurology, Columbia University Irving Medical Center, New York, New York, USA

^4^ Department of Neurology, University of Colorado, Aurora, CO, USA

^5^ The Michael J. Fox Foundation for Parkinson’s Research, New York, New York, USA

^6^ Department of Neurology, Beth Israel Deaconess Medical Center, Boston, MA, USA

^7^ JPG Enterprises LLC, Chicago, Illinois, USA

^8^ National Institute of Neurological Disorders and Stroke, National Institutes of Health, Bethesda, Maryland, USA

^9^ Department of Neurology, University of California, Irvine, CA, USA

^10^ Department of Molecular and Medical Genetics, Oregon Health & Science University, Portland, Oregon, USA

^11^ Department of Neurology, Department of Neurology, Boston University School of Medicine, Boston, Massachusetts, USA

^12^ Department of Neurology, University of Minnesota, Minneapolis, Minnesota, USA

^13^ Parkinson Disease and Movement Disorder Program, Department of Neurology, University of Chicago, Chicago, Illinois, USA
